# Origin of the anomalous size-dependent increase of capacitance in boron nitride–graphene nanocapacitors

**DOI:** 10.1039/c9ra00614a

**Published:** 2019-03-11

**Authors:** Orion Ciftja

**Affiliations:** Department of Physics, Prairie View A&M University Prairie View TX 77446 USA ogciftja@pvamu.edu

## Abstract

The anomalous size-dependent increase in capacitance in boron nitride–graphene nanocapacitors is a puzzle that has been initially attributed to the negative quantum capacitance exhibited by this particular materials system. However, we show in this work that the anomalous nanocapacitance of this system is not due to quantum effects but has pure electrostatic origin and can be explained by a parallel-plate (square) nanocapacitor model filled with a dielectric film characterized by a size/thickness-dependent relative permittivity. The model presented here is in excellent agreement with the experimentally measured capacitance values of recently fabricated graphene and hexagonal boron nitride nanocapacitors. The results obtained seem to suggest that the size-dependent increase of capacitance in the above-mentioned family of nanocapacitors can be explained by classical finite-size geometric electrostatic effects.

## Abstract 2

The anomalous size-dependent increase in capacitance in boron nitride–graphene nanocapacitors is a puzzle that has been initially attributed to the negative quantum capacitance exhibited by this particular materials system.

## Graphical abstract

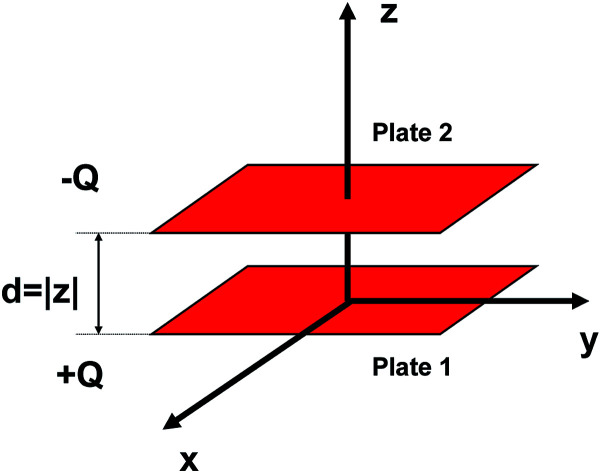

## 1 Introduction

Experiments have typically shown that the reduction of capacitance at small sizes is due to quantum effects that originate from a positive quantum capacitance (*C*_q_ > 0) associated with a “dead-layer” quantum effect. However, as found out in a recent study^1^ this would not be the case for materials that show a negative quantum capacitance, *C*_q_ < 0. For some materials, negative quantum capacitance may lead to a significant increase of the capacitance as thickness decreases, a trend that is consistent with the conventional wisdom based on electrostatic principles. In particular, it was predicted that an ultrathin nanocapacitor made of graphene electrodes (plates) filled with hexagonal boron nitride (h-BN) dielectric nanofilm can achieve superior capacitor properties with a significant increase of the capacitance with reduction of thickness below a certain value.^1^ This anomalous size dependence of nanocapacitance (labeled “anomalous” because it is inconsistent with the expected “dead-layer” quantum effect) appears when the thickness becomes less than circa 5 nm, that is less than 16 h-BN atomic layers. At this setup, the relative permittivity of the dielectric film systematically increases with decreasing h-BN thickness^1^ and is different from the bulk BN value. This anomalous size-dependent increase in capacitance was attributed to a negative quantum capacitance exhibited by this particular system of materials.

In this work we introduce a model for a parallel-plate nanocapacitor^2^ consisting of two plates placed face-to-face at an arbitrary distance apart with all spatial dimensions and/or inter-plate separation distance constrained to be in the nanoscale. For simplicity, each of the plates of the nanocapacitor is assumed to be a perfectly two-dimensional (2D) square with a length, *L* that measures on the nanoscale. We do not consider any quantum effects in our model. The influence of a dielectric material film between the plates of the nanocapacitor^3–5^ is accounted for in the model through the inclusion of a phenomenological size/thickness-dependent relative permittivity parameter with a value different from that of the bulk. Theoretical nanocapacitance values obtained from this model reproduce the experimental results for boron nitride–graphene capacitors and lead us to believe that the anomalous size-dependent increase of capacitance in boron nitride–graphene nanocapacitors cannot be attributed to quantum effects.

## 2 Theory

**Fig. 1:**
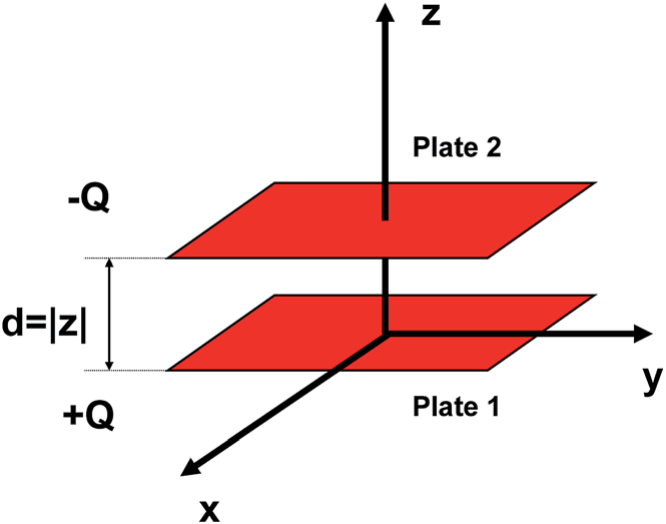
Schematic view of a parallel-plate nanocapacitor consisting of two identical plates, 1 and 2 placed face-to-face a distance, *d* = |*z*| apart. The two plates of the nanocapacitor are assumed to be identical squares with length, *L*. The plates contain, respectively, a total charge of ±*Q* which is considered uniformly spread over the surface area resulting in a uniform surface charge density, ±*Q*/*L*^2^.

We consider a parallel-plate nanocapacitor consisting of two identical uniformly charged plates 1 and 2 at an arbitrary separation distance. For simplicity, we assume that the two plates of the nanocapacitor are squares with an arbitrary length, *L*. The plates contain, respectively, a total amount of charge of +*Q* and −*Q* that is considered to be uniformly spread over the corresponding surfaces. Since the two square plates are identical, their respective uniform surface charge densities are: ±*Q*/*L*^2^. To simplify the notation we denote by *σ* the surface charge density of the positively charged plate, *σ* = +*Q*/*L*^2^. We choose a Cartesian system of coordinates so that the two plates lie parallel to each other and perpendicular to the *z*-direction. The origin of the Cartesian system of coordinates is chosen at the center of plate 1. The *z* axis is taken perpendicular to its plane while the *x* and *y* axes are parallel to its edges. For this choice of the coordinative system, plate 1 of the nanocapacitor lies in the *x*–*y* plane at *z* = 0 while plate 2 lies in the *x*–*y* plane at some arbitrary *z*. Any two arbitrary elementary charges, *dq*_1_ and *dq*_2_ interact with each other *via* a standard Coulomb interaction potential, *k*_e_*dq*_1_*dq*_2_/|*r⃑*_1_ − *r⃑*_2_| where *k*_e_ is Coulomb's electric constant. Fig. 1 gives a schematic presentation of the nanocapacitor system under consideration.

The immediate objective of the study is to calculate exactly the total electrostatic energy stored in this nanocapacitor and derive its nanocapacitance from that expression. The nanocapacitance values obtained from the model will be compared to corresponding experimental results for the family of boron nitride–graphene nanocapacitors under consideration. The total electrostatic energy of the nanocapacitor can be written as:

1 *U* = *U*_11_ + *U*_22_ + *U*_12_ = 2*U*_11_ + *U*_12_,

where *U*_11_ (*U*_22_) represent, respectively, the Coulomb self-energy of plate 1 (2) while *U*_12_ represents the Coulomb interaction energy between the two oppositely charged plates of the nanocapacitor. It is obvious from basic considerations that *U*_11_ = *U*_22_. The Coulomb self-energy of a uniformly charged square plate, namely, the term *U*_11_ has been calculated and the method is described in detail in ref. 6 and 7 with the final result written as:2
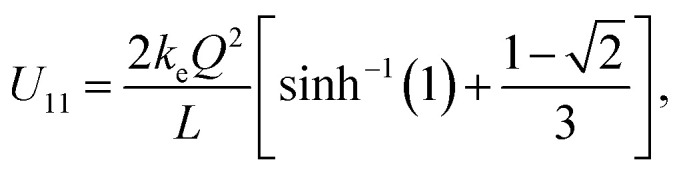
where 
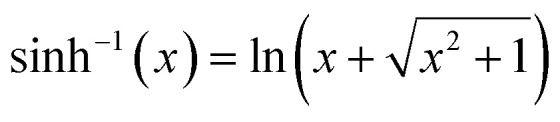
 is the inverse hyperbolic sine function.

The approach used to calculate the self-energy, *U*_11_ can be adopted to obtain the interaction energy term, *U*_12_. The final exact expression for the interaction energy between the two parallel plates of the nanocapacitor reads:3
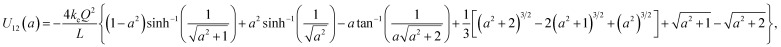
where, from now on, the parameter, *a* = *z*/*L* is specifically shown in the argument of the interaction energy function and tan^−1^(*x*) denotes an inverse tangent trigonometric function. The lengthy mathematical details of the calculations that lead to the result above are thoroughly explaned in ref. 8.

Substitution of the results from eqn (2) and (3) into eqn (1) leads to the following exact analytical expression for the total energy stored in a parallel-plate (square) nanocapacitor with free space between the plates:4
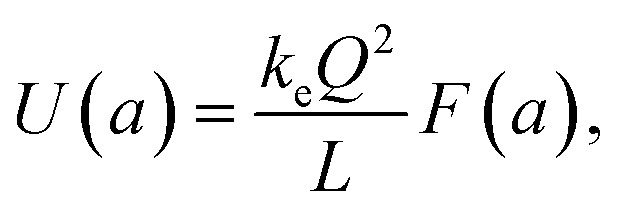
where5
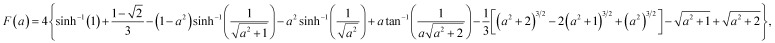
and the parameter *a* = *z*/*L* is explicitly shown in the argument of the total energy function. As deduced from eqn (5), one can replace *a* with |*a*| in all expressions above since *a*^2^ = |*a*|^2^. One can, again, verify, starting from the general expression in eqn (5), that

6 *U*(*a* = 0) = 0.

We note that, unlike the case of point charges, the Coulomb interaction energy between any two bodies with uniform charge distributions in two or three dimensions is not infinite when the two bodies overlap. See, for example, the value of the interaction energy of a pair of identical coplanar uniformly charged nanodisks as reported in eqn (14) of ref. 9 or, similarly, the finite Coulomb self-energy result in eqn (2) of the current work. It should be noticed from the definition of the Coulomb energy terms in integral form that *U*_12_(*a* = 0) = −2*U*_11_. This means that the negative energy of the attracting plates is exactly twice (and opposite) to the positive Coulomb self-energy of a given plate when the two plates of the capacitor overlap (at *a* = 0). This leads to a full cancelation of all the energy terms as in eqn (6). This is also consistent with the expectation that, in such a limit, the positive charge cloud, +*Q* and the negative charge cloud, −*Q* would annihilate each other.

Therefore, one can obtain the dependence of *U*(*a*) for small *a* by expanding the function in eqn (5) around *a* = 0. We found the following expression valid to the lowest order (linear order) of parameter, *a*:7
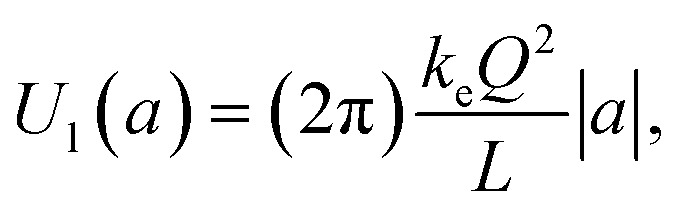
where |*a*| = |*z*|/*L* and |*z*| is the separation distance between the two plates of the parallel-plate nanocapacitor. The key mathematical details of how eqn (6) and (7) are derived from the expression in eqn (5) are provided in the Appendix A.

**Fig. 2:**
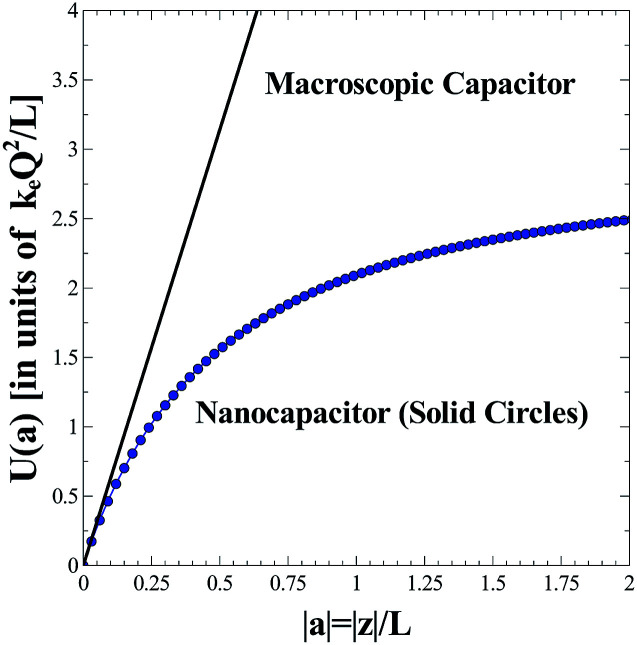
Energy stored in a parallel-plate nanocapacitor, *U*(*a*) in units of *k*_e_*Q*^2^/*L* as a function of the parameter |*a*| = |*z*|/*L* (solid circles) where |*z*| is the separation distance between the two parallel plates placed face-to-face. The shape of the plates is assumed to be square with a finite length *L*. The plates contain, respectively, a charge of ±*Q* that is uniformly spread over the surface. The exact result obtained is compared to the approximate expression, *U*_l_(*a*) that represents the stored energy for the case of a macroscopic bulk ideal parallel-plate capacitor (solid line).

In Fig. 2 we plot the energy stored in a parallel-plate nanocapacitor, *U*(*a*) as a function of |*a*| = |*z*|/*L* (solid circles) and compare it to the approximate linearized energy potential, *U*_l_(*a*) (solid line). The energies are expressed in units of *k*_e_*Q*^2^/*L*. One can prove that *U*_l_(*a*) represents the energy stored in a macroscopic ideal parallel-plate capacitor constructed from two infinite uniformly charged plates of area *A* in free space:8
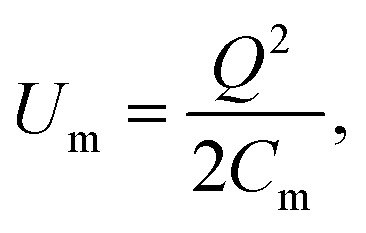
where (in free space), 
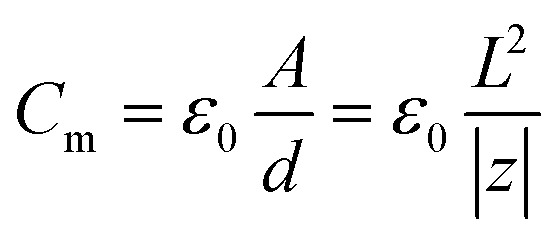
.

The equivalence of *U*_l_(*a*) in eqn (7) to *U*_m_ in eqn (8) is easy to verify if one starts from eqn (8) and rewrites it in terms of *k*_e_*Q*^2^/*L* and |*a*| = |*z*|/*L*. One must not forget that Coulomb's electric constant is *k*_e_ = 1/(4π*ε*_0_). As seen from Fig. 2, the energy stored in a finite-size nanocapacitor as calculated from eqn (5) differs substantially from the value obtained when the bulk formula of a macroscopic ideal parallel-plate capacitor is used [see eqn (7)] even for separation distances of the order of |*z*|/*L* ∝ 10%. Within the framework of this model, one would account for the presence of a dielectric material between the plates by introducing a phenomenological size/thickness-dependent relative nanopermittivity parameter in the expression for the energy stored. The nanopermittivity of very thin dielectric films is expected to be different from the bulk value and is obtained experimentally as a function of size and/or thickness.

## 3 Nanocapacitance

The energy expression in eqn (8) rewritten for the case of a nanocapacitor leads to an analytical result for the corresponding size-dependent geometric nanocapacitance:9
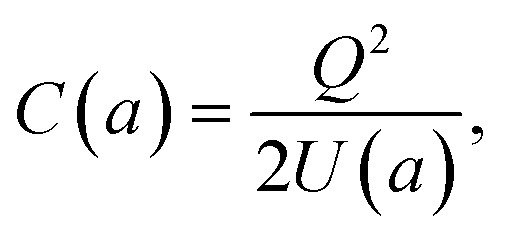
where *U*(*a*) is given from eqn (4) and (5). One uses eqn (4) and (5) to write *C*(*a*) in a more convenient form as:10
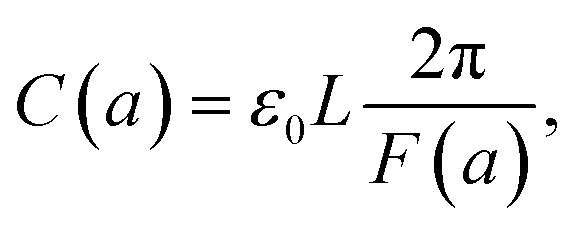
where *ε*_0_ ≈ 8.85 × 10^−12^ F m^−1^ (F = Farad) and the function *F*(*a*) is given from eqn (5). Note that the expression in eqn (10) would apply to a nanocapacitor without a dielectric between the plates. A straightforward generalization of the formula in eqn (10) to a more realistic situation where a dielectric material is inserted between the plates of the nanocapacitor reads:11
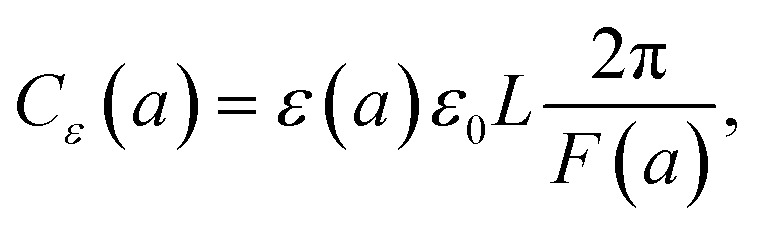
where *ε*(*a*) represents the relative nanopermittivity of the dielectric film expected to be size/thickness-dependent and different from the bulk value of that material. Note that in this model, the role played by a dielectric film in the nanocapacitor (when taken into account) is included in a conventional way by adding a phenomenological size/thickness-dependent relative nanopermittivity parameter in the expression for the capacitance. This means that we do not take into consideration any of the very subtle quantum effects (such as the “dead-layer” effect, *etc.*) that have been seen in a variety of nanocapacitors dominated by quantum effects. On the contrary, we believe that the origin of the anomalous size-dependent increase of capacitance in boron nitride–graphene nanocapacitors is not quantum.

Nevertheless, considering the importance of quantum effects in several other nanosystems, we briefly discuss some known characteristics of what would be a typical quantum behavior in a nanacapacitor. To this aim, we point out again that one of the most significant quantum effects that happen in capacitive systems is the so-called “dead-layer” effect, namely, the appearance of two atomically thin layers (that can be modeled as a 2D charged system) that modify the net capacitance to:12
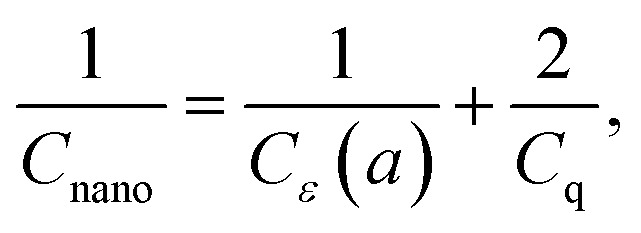
where *C*_*ε*_(*a*) represents the capacitance assuming the dielectric fills the space in between the plates without any quantum effects and *C*_q_ is the additional quantum capacitance. A determination of the value of *C*_q_ is not trivial and, likewise, it is not easy to model the electric properties of a dielectric nanofilm consisting of only a few atomic layers. For such a case, one must use an effective nanopermittivity, *ε*_nano_ = *ε*(*a*) different from the bulk value and an effective thickness parameter, *d*_nano_ different from the geometric thickness, *d* (namely, effective *d*_nano_ ≤ *d*). In a nutshell, the dielectric medium has an important contribution in capacitative properties and the relative permittivity values of the dielectric film are size (thickness) dependent. As explained earlier in the follow up discussions to eqn (11) these important aspects are considered in our model for the nanocapacitance. The effect of the dielectric film in the overall capacitance of the nanocapacitor is accounted for through the inclusion of a phenomenological size/thickness-dependent relative nanopermittivity parameter in the expression for the capacitance. The values of such a parameter for various thicknesses are determined experimentally, for instance, see ref. 1 for the case of h-BN/graphene nanocapacitors.

The model in eqn (11) may be a reasonable approximation to all experimental situations where the capacitance of a nanocapacitor is dominated by geometric effects, namely, when it is noticed that a decrease of the thickness of the dielectric film leads to an increase of the capacitance. Experiments in a nanocapacitor made of graphene plates/electrodes and h-BN dielectric films show an increase of capacitance with decrease of thickness.^1^ Since this pattern is shared by the geometric capacitance of the model in eqn (11) we checked if such a model applies to these experiments. We specifically took into consideration the experimental data from seven groups of different h-BN/graphene nanocapacitors that have the Au/h-BN/Au stack structure.^1^ The graphene electrodes are treated as square plates with length, *L* = 10 μm (area 100 μm^2^). For such a case, one calculates that:13
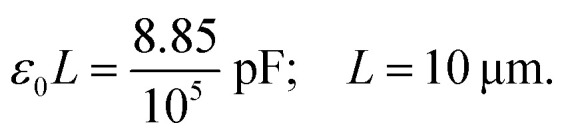


We calculated the resulting values of the capacitance using the theoretical model in eqn (11) where all the values of various experimental parameters are taken from Table 1 of ref. 1. The results are shown in Table 1. The theoretical values of the capacitance obtained from our model in eqn (11) are in excellent agreement with the experimental data (except one data point). The only experimental data that is not in perfect agreement with the model in eqn (11) appears to be an anomaly (experimental value of 0.735 pF *versus* theoretical value of 0.538 pF). These results seem to suggest that the net capacitance of this class of h-BN/graphene nanocapacitors^1^ is dominated by geometric classical electrostatic effects. This would explain the increase of the capacitance as thickness decreases since such a behavior is consistent with conventional electrostatic principles.

**Table 1:** Parallel-plate nanocapacitor consisting of a square plate with length, *L* = 10 μm (area 100 μm^2^). The quantity, |*z*| represents the separation distance (thickness) between the two plates. The nanocapacitor is filled with a dielectric film that has a size/thickness-dependent relative permittivity, *ε*(*a*) that is a function of the dimensionless quantity, |*a*| = |*z*|/*L*. The theoretical value of the capacitance, *C*_*ε*_(*a*) obtained from the model in eqn (11) is compared to the experimental data.^1^ The experimental data are from seven groups of different h-BN/graphene nanocapacitors that have the Au/h-BN/Au stack structure where the values of *L*, *z* and *ε*(*a*) are the same as the one reported in ref. 1

*L*	|*z*|	|*a*| = |*z*|/*L*	*ε*(*a*)	Capacitance *C*_*ε*_(*a*) [theory]	Capacitance [experiment]
10 μm	26.7 nm	26.7 × 10^−4^	4.2	0.141 pF	0.139 pF
10 μm	20.2 nm	20.2 × 10^−4^	4.2	0.186 pF	0.184 pF
10 μm	14.5 nm	14.5 × 10^−4^	4.2	0.258 pF	0.256 pF
10 μm	8.1 nm	8.1 × 10^−4^	4.9	0.538 pF	0.735 pF
10 μm	2.8 nm	2.8 × 10^−4^	5.6	1.773 pF	1.770 pF
10 μm	1.5 nm	1.5 × 10^−4^	6.8	4.016 pF	4.012 pF
10 μm	0.8 nm	0.8 × 10^−4^	7.9	8.744 pF	8.739 pF

## 4 Conclusions

We introduced a model for a parallel-plate nanocapacitor consisting of two square plates uniformly charged equally but oppositely. The expressions in eqn (4) and (5) constitute the exact final result for the total energy stored in a parallel-plate nanocapacitor consisting of two square plates with arbitrary area, *L*^2^ placed at an arbitrary separation distance, |*z*|. The exact analytic formula derived in eqn (5) is useful to understand how energy is stored in multi-layered capacitive nanostructures that interact electrostatically.^10–13^ The expression for the energy stored in a parallel-plate nanocapacitor can be readily used to derive an analytic formula for the capacitance of such a nanocapacitor model. When considering a nanocapacitor with a dielectric film between the plates, one assumes a phenomenological size/thickness-dependent relative nanopermittivity for the dielectric material that is not the same as the bulk value. It was found that the theoretical model for the nanocapacitance introduced in eqn (11) is in excellent agreement with the experimental capacitance measurements of a novel class of nanocapacitors made of graphene and ultrathin h-BN dielectric films.^1^

Within the framework of classical electrostatics, the size of the plates and the distance between the plates defines the value of the capacitance for a given dielectric medium between the plates. Our work finds out that a nanocapacitor of graphene plates filled with a h-BN dielectric film manifests capacitance that is consistent with conventional electrostatics. The results obtained strongly suggest that the size-dependent increase of capacitance with size reduction in the family of boron nitride–graphene nanocapacitors is due to the geometric capacitance of the system and can be explained by classical finite-size geometric electrostatic effects.

## A Mathematical derivations

Let's rewrite the quantity in eqn (5) as a function *F*(*x*) of the form:14
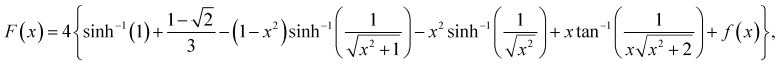
where *f*(*x*) is a new auxiliary function:15
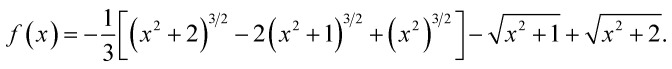


It is assumed that *x* ≥ 0 and real (since after all, *x* represents |*a*|). One can verify the truthfulness of eqn (6) if one can show that *F*(*x* = 0) = 0. To see that, one relies on the following formulae for two relevant limits:16
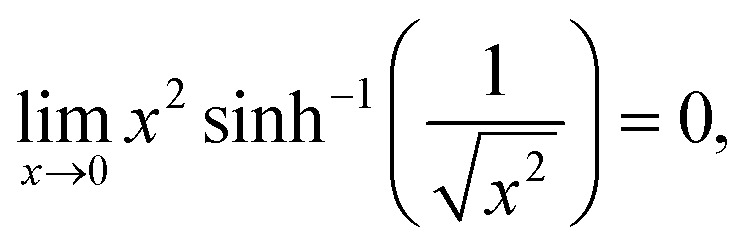
and17
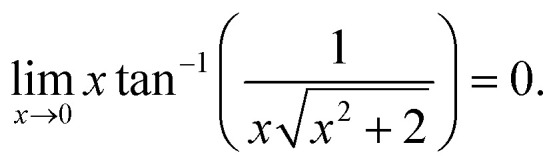


One can easily check that *F*(*x* = 0) = 0 based on the results from eqn (16) and (17) since all the other terms in the expression for *F*(*x*) are easy to calculate for *x* = 0. Note that 
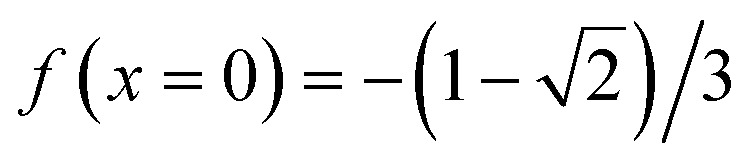
.

To see the dependence of *F*(*x*) to the lowest order of *x*, we expand *F*(*x*) around *x* = 0 and keep terms up to the quadratic power of *x*. The following small-*x* expansions apply:18
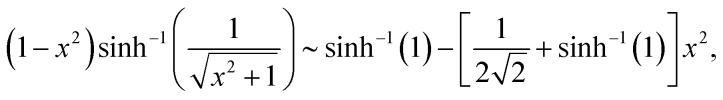
19
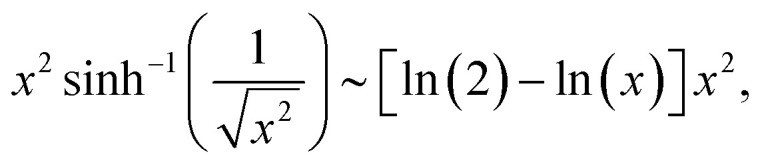
20
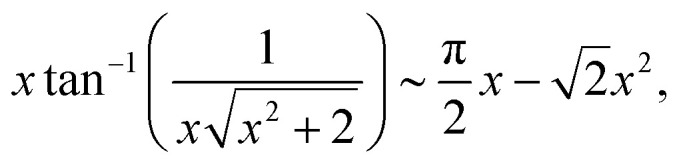
and21
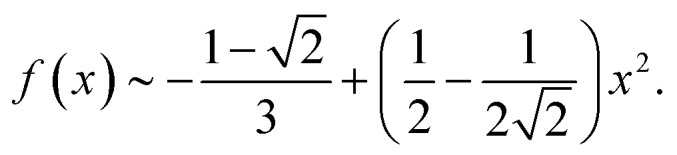


By putting together all the results above in the expression for *F*(*x*) and keeping the lowest order of *x* (linear order), one eventually obtains:

22 *F*(*x*) = 2π*x* + 0(*x*^2^).

This result is used to derive the expression for the energy shown in eqn (7).

## Conflicts of interest

There are no conflicts to declare.

## Supplementary Material

## References

[cit1] Shi G., Hanlumyuang Y., Liu Z., Gong Y., Gao W., Li B., Kono J., Lou J., Vajtai R., Sharma P., Ajayan P. M. (2014). Nano Lett..

[cit2] Engheta N., Salandrino A., Alù A. (2005). Phys. Rev. Lett..

[cit3] Kim Y., Han H., Vrejoiu I., Lee W., Hesse D., Alexe M. (2011). Appl. Phys. Lett..

[cit4] Ekanayake S. R. , Ford M. and Cortie M., Materials Forum, Institute of Materials Engineering Australasia, 2004, vol. 27, p. 15

[cit5] Li L.-J., Zhu B., Ding S.-J., Lu H.-L., Sun Q.-Q., Jiang A., Zhang D. W., Zhu C. (2012). Nanoscale Res. Lett..

[cit6] Ciftja O. (2010). Phys. Lett. A.

[cit7] Ciftja O. (2016). Adv. Math. Phys..

[cit8] Ciftja O. (2016). J. Nanosci. Nanotechnol..

[cit9] Ciftja O., Berry I. (2018). AIP Adv..

[cit10] Akbar S., Lee I.-H. (2001). Phys. Rev. B: Condens. Matter Mater. Phys..

[cit11] Ishizuki M. I., Takemiya H., Okunishi T., Takeda K. (2012). Phys. Rev. B: Condens. Matter Mater. Phys..

[cit12] Califano M., Harrison P. (1999). J. Appl. Phys..

[cit13] Welander E., Burkard G. (2012). Phys. Rev. B: Condens. Matter Mater. Phys..

